# Experiences and Supportive Care Needs of Latinx Millennial Caregivers

**DOI:** 10.1177/10436596241274116

**Published:** 2024-09-13

**Authors:** Catie Cleary, Galilea Dupree, Anna Welling, Janice F. Hernandez, Heather Cuevas, Michael Thomas, Neil Peterson, Sharon D. Horner, Megan Thomas Hebdon

**Affiliations:** 1 The University of Texas at Austin, USA; 2 Brigham Young University, Provo, UT, USA

**Keywords:** family caregiver, Latinx, chronic disease, Millennial

## Abstract

**Introduction:**

Latinx Millennial caregivers are an understudied minority group in the United States. Due to life stage and cultural values, these caregivers struggle to balance conflicting priorities with career, family, and caregiving. They also face systemic barriers and healthcare disparities.

**Methods:**

Participants (*N* = 29) were recruited locally and nationally. Qualitative data were collected using five focus groups and one individual interview. Interviews were analyzed by seven coders using thematic analysis with an inductive approach.

**Results:**

Meta-themes included (a) the Latinx experience with culture, immigrant status, and structural barriers; and (b) being a super caregiver: being everything to everyone. Additional main themes were identified including family well-being, occupational and financial well-being, social support dynamics, challenges and rewards of family caregiving, and coping strategies.

**Discussion:**

Clinical interventions for Latinx Millennial caregivers should address cultural background, value of family/community, and systemic barriers for care and support.

Twenty-seven percent of Millennial caregivers (born between 1981 and 1996) identify as Hispanic/Latinx, and they are the largest ethnic minority subgroup within the Millennial caregiving cohort (Flinn, 2018; National Alliance for Caregiving [NAC] & AARP, 2020). Due to age (early-middle adulthood) and life stage, Latinx Millennial caregivers manage competing priorities of career, family, and caregiving while navigating the challenges of being an ethnic minority in the United States (Bialik & Fry, 2019; Flinn, 2018; Franssen et al., 2020; Velasco-Mondragon et al., 2016). In early-middle adulthood, individuals are often focused on achieving important milestones such as marriage or intimate partnerships, childbearing, establishing financial independence, and pursuing long-term employment and/or career development (Infurna et al., 2020). For Latinx caregivers, family caregiving responsibilities are more complex due to higher rates of caring for individuals with multi-morbidity (Doose et al., 2023; Quiñones et al., 2019; Rote et al., 2019). Balancing heavy caregiving responsibilities and systemic barriers to care with developmental and financial milestones of early-middle adulthood places this group at unique risk for increased stress and its resulting physical and mental health challenges (Moody’s Analytics, 2019; Velasco-Mondragon et al., 2016). This study seeks to understand the specific challenges and demands of Latinx Millennial caregivers to better design interventions that meet their needs.

Latinx Millennial caregivers (34%) are more likely to be higher-hour caregivers (providing care for more than 20 hr/week) than their African American/Black (29%) or White (20%) counterparts (Flinn, 2018). In addition, Latinx Millennial caregivers are more likely to work outside the home and work longer hours per week than White or Black/African American caregivers (NAC & AARP, 2020). As a result, the stress of managing the competing priorities of work and family caregiving is amplified for Latinx Millennial caregivers, who have less time each week for self-care activities to help them cope with these competing stressors (Guidi et al., 2021; Meyer, 2003; Pearlin et al., 1990). To add to this stress, Latinx individuals living in the U.S. are disproportionately negatively affected by social determinants of health. This results in health inequities, including decreased access to healthcare (National Academies of Sciences, Engineering, and Medicine, 2017; Velasco-Mondragon et al., 2016).

## Gender and Cultural Context

Latinx Millennial caregivers are more evenly split by gender with 57% of Latinx Millennial caregivers identifying as men (Flinn, 2018). This contrasts with the literature that has described that first-born Latinx women feel greater pressure to take on the primary family caregiving role (Longoria et al., 2020). Machismo (masculinity) and marianismo (femininity) are gender scripts within Latinx culture (Badger et al., 2019; Nuñez et al., 2016). These roles may be more dynamic and complex within individuals and there may be shifts in how gender roles operate in younger generations with family caregiving (Nuñez et al., 2016; Velasco-Mondragon et al., 2016). This may be due to how gender roles are internalized and enacted, the degree of acculturation for individuals, and the spread of family caregiving across multiple individuals within a family system (National Alliance for Caregiving [NAC], 2019; Nuñez et al., 2016; Rote et al., 2019; Velasco-Mondragon et al., 2016). In addition, caballerismo, the counterpart to machismo, focuses more on nurturing, chivalry, and family centeredness for a masculine identity (Arciniega et al., 2008). This may be an additional lens to view Latinx family caregiving for Millennial men.

In addition to structural challenges, Latinx Millennial caregivers have a cultural context with protective factors and stressors. *Familismo*, or familism, refers to the Latinx cultural norm of loyalty to one’s family, communalism, reciprocity, and interdependence between nuclear and extended family networks (Badger et al., 2019; Sabogal et al., 1987; Velasco-Mondragon et al., 2016). This perpetuates the collectivist expectation of caring for one’s family members at the cost of meeting their individual financial, emotional, and physical needs (Gelman, 2014; Mendez-Luck, Applewhite, et al., 2016). For some family caregivers, familismo can serve as a protective factor for health outcomes such as substance abuse, mental health, and positive well-being (Brooks et al., 2014; Gil et al., 2000; Harker, 2001). For example, population-level data show higher caregiver hours and more complex caregiving in Latinx family caregivers, yet these caregivers report less emotional stress, financial burden, and overall family caregiving burden (Flinn, 2018; National Alliance for Caregiving & AARP, 2020). Compared with their White counterparts, Latinx family caregiving may be shared among multiple individuals, rather than a primary family caregiver (NAC, 2019; Rote et al., 2019). Familismo may vary based on individual acculturation (Falzarano et al., 2022), and has more complex applications to individual experiences of family caregiving than is often acknowledged in research and practice (Gelman, 2014). Spirituality is a core value within Latinx culture (Badger et al., 2019). This is more complex and individualized among Latinx family caregivers, particularly in the Millennial generation where alignment with a religious tradition is declining (Pew Research Center, 2015). Spiritual practice or openness to spiritual experience may be increasing, which is another area of resilience for Latinx Millennial caregivers (Badger et al., 2019; Gallegos & Segrin, 2019; Pew Research Center, 2015; Thomas Hebdon et al., 2023).

### Stress Process Model and Latinx Millennial Caregivers

**Figure 1.**
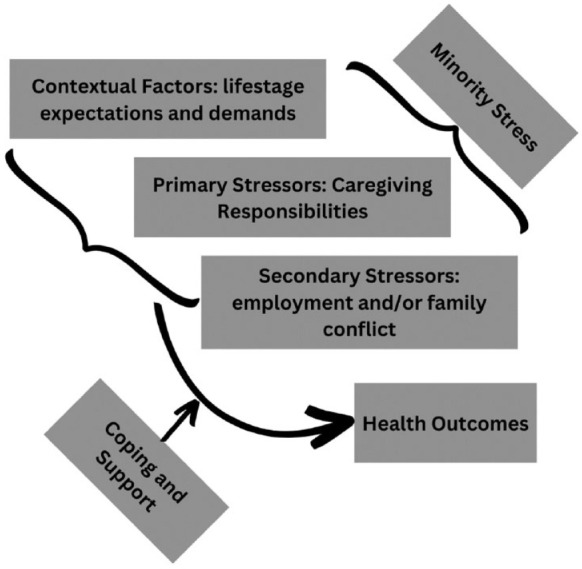
Stress Process Model for Latinx Millennial Caregivers.

The stress response of Latinx Millennial caregivers (Figure 1) can be understood through the Stress Process Model (Pearlin et al., 1990). Stress is the result of a complex set of interrelated conditions, including primary stressors (caregiving responsibilities), secondary stressors (employment and/or family conflict), and contextual factors (life stage expectations and demands). While stressors can result in physical and mental illness, individuals may avoid the worst of these effects and enjoy better health outcomes by developing coping skills and receiving adequate social support (Pearlin et al., 1990). An important overlay to the Stress Process Model specifically for the Latinx population is Minority Stress Theory (Meyer, 2003). In Minority Stress Theory, individuals who identify within minority groups experience added social stressors related to prejudice, stigma, and discrimination. These stressors experienced due to prejudice, stigma, and discrimination are chronic, and they originate from the social environment (Meyer, 2003). Therefore, Latinx Millennial family caregivers are experiencing the stress of family caregiving and other life demands in conjunction with minority stress (Velasco-Mondragon et al., 2016). This could intensify the stress experience and put these individuals at greater risk for long-term health consequences, particularly if coping, support, and other interventions are not in place to reduce the caregiving load (Guidi et al., 2021; Meyer, 2003; Pearlin et al., 1990). The purpose of this qualitative descriptive study was to explore the experiences and needs of Latinx Millennial caregivers with the aim of designing future interventions to support them in stress management and support access.

## Method

### Study Design and Participants

This was the first phase of a three-phase multi-method study examining the needs and experiences of Latinx Millennial caregivers to adapt and refine an mHealth intervention for this population. This phase is a qualitative descriptive analysis of focus group interviews to understand the experiences and needs of Latinx Millennial caregivers. Findings from the other phases are reported elsewhere.

Participants (*N* = 29) were recruited using purposive sampling through community settings, professional contacts, national caregiving groups, and online means through a combination of paid social media advertisements and informal advertising within online family caregiving support group platforms and community clinics in Texas and Utah. Participants filled out an eligibility survey via REDCap, which confirmed that they were born between 1981 and 1996, identified as Latinx, and provided care to a family member or friend for at least 10 hr per week. Participants were excluded if they did not have reliable internet access given that focus groups were conducted over Zoom.

### Data Collection and Analysis

Eligibility survey data was collected through REDCap electronic data capture tools hosted by The University of Texas (Harris et al., 2009, 2019). Participants indicated if they were willing to participate in an online focus group in the eligibility survey. Willing and eligible participants were contacted over email by a graduate research assistant, who provided participants with a link to a Zoom meeting. One researcher (MH) conducted five Zoom focus groups of 60-90 min with between 3 and 10 individuals (see Table 1). A research assistant (CC) independently recorded observations in a separate document during the focus group meetings. One individual participant interview occurred due to the participant being unavailable to participate in the focus groups. Focus groups and the interview were recorded and transcribed verbatim. Focus groups were sequentially held until accumulation of sufficient data for saturation and inductive thematic saturation, where data generated during the focus groups became redundant and new themes/codes were not encountered during data analysis (Saunders et al., 2018). The focus groups and interview were analyzed concurrently, and there was high agreement in themes among all data sources. Participants were provided with a US$70 gift card for participating in the focus groups or interview. Upon completion of the focus group, participants were also sent a demographic survey, administered through REDCap, with a combination of closed- and open-response questions pertaining to their demographic background and caregiving experiences.

**Table 1.** Sample Focus Group Questions.

Caregiving experiences
To get us started, can each of you tell us about your caregiving situation? We also want to know about the other things in your life you are doing besides caregiving. Thank you so much for what you have shared. Now, can you share what is most challenging about caregiving for you? We also like to know about the good things that come from caregiving. Can you share any positive things that have come from your caregiving?
Support needs
Can you share what support you have received that has been helpful? Please describe any support you have received that was not helpful. Now we want to know if there is support you feel like you need that you have not received?

Demographic data were analyzed for frequencies and descriptive statistics. Data from the focus groups and interview were analyzed inductively by seven coders (the principal investigator, four co-investigators, and two student research assistants) using qualitative thematic analysis and the Dedoose software (Braun & Clarke, 2006; Dedoose Version 9.0.17., 2021). The focus groups and interview were coded by all coders, with the goal of integrating multiple perspectives into the coding process for coder triangulation (Lincoln & Guba, 1985). Coders addressed their positionality throughout the coding process: two of the team members identify as family caregivers, four identify as Millennials, and three identify as Latinx. Discussion of identity and how this impacts data analysis occurred to ensure that coders remained close to the data and reflected what participants communicated, rather than what their perspectives communicated (Lincoln & Guba, 1985). Themes, sub-themes, and definitions were reviewed and refined during each team meeting. The principal investigator moderated discussions and compiled coding for final review and interpretation. Exemplar quotes were selected from the data and incorporated into a table (Table 2) to illuminate the experiences of participants. Notes were kept during each coding meeting and shared with the research team during weekly team meetings. Confidentiality was maintained by storing all study data such as notes, focus group transcripts, and thematic analysis within a collaborative secure cloud storage accessible solely by research team members.

**Table 2:** Experiences and Supportive Care Needs of Latinx Millennial Caregivers: Themes and Sub-Themes.

Overarching themes		
Latinx experience: Caregiving experiences specific to being a Latinx family caregiver including immigration experiences, *familismo*, community, being minoritized: “. . . my mom had to be in hiding before she was able to get her green card” (P1I1). “. . . we’re a very large Hispanic family” (P1G2). “He worked very hard all his life and . . . you guys aren’t showing him any kind of respect” (P1G2).		
Super caregiver: Caregivers must “do it all” “I have to do the caring for everyone, and most times I tend to overlook myself” (P1I1). “I just have to keep thinking about you know the stress isn’t really in the fact that. You have to do things for them for the people you take care of because most times you’re willing to do those things and you know, once you have the will, the motivation, it all becomes easier” (P5G2).		
Theme and definition	Sub-theme	Exemplar quote
Family well-being *The need to promote family well-being through their family caregiving responsibilities as well as the relationships and responsibilities they have with other family members.*	Importance of family relationships	“I’m supposed to take the kids out even though I’m not able to spend a full day with my kids . . . maybe go on game day with them” (P1G1).
	Balancing time and attention with family and caregiving	“I don’t have much time to spend with my friends as much as I would want to because I try my best to get back to my dad and kids from work as quickly as possible” (P1G5).
	Challenges maintaining intimate partner relationships	“Maybe take my wife out for dinner, or maybe try to make her feel special . . . just to make sure the marriage is going on smoothly” (P1G1).
Occupational and financial well-being *The experiences and challenges of maintaining employment and meeting the financial demands of family caregiving and daily life.*	Balancing living costs with caregiving costs	“So, it’s not really easy, because sometimes I have to work like two to three jobs to make it possible . . . people need insulin” (P1I1).
	Cost of medication and healthcare	“I have to do it myself to save money and to be able to get her medications too” (P1I1).
	Daily living costs	“I’ve been taking care of my grandma for five years now and during the pandemic, it was really, really hard, I mean you know food got more expensive” (P5G2).
	Workplace support and flexibility	“My work is mainly online so it will not interfere with caregiving with my granddad. So, I can handle both caregiving and my job . . . it doesn’t really interfere with it, that much . . . yeah, it’s really flexible” (P3G4).
	Absenteeism, presenteeism, leaving the workforce	“I actually don’t have a full concentration on what I’m doing, most of the time I sleep at work” (P2G1).
Social support dynamics *The experiences of family caregivers in receiving or needing social support from structural systems (government, healthcare, etc.), family, and other caregivers as well as the educational, emotional, and financial support needed to be a family caregiver.*	Needing structural support	“I know we would feel better supported if we had more financial support and kind of had a recognition from the government . . . our needs would be better provided” (P1G5).
	Family support	“They know it’s not easy, and they know I’m doing them a favor [by] taking care of their mom. It makes my caregiving bearable, because it would be really stressful without the support from them” (P3G5).
	Connections with other caregivers	“Sometimes I just need a community where I can take my grandma. Where you’ll just get together and recognize yourself as caregivers and encourage yourself emotionally and laugh too” (P2G1).
	Specific support needed: Educational, emotional, and financial support	Educational: “What I think is missing is that maybe ways we can plot the stages of our person we care for . . . for example, in the chronic illness. There are stages with it may be mild, moderate or severe” (P3G1). Emotional: “I didn’t know the emotional and mental drain it was going to put on me when I took on the task. Like I wish there was a resource to help you sort through your emotions without feeling guilty about it. You can’t really do that with the person you’re helping because it makes them feel bad” (P2G4). Financial: “Caregiving can also be really expensive, so financial support would be helpful as well” (maybe P1G2).
Challenges and rewards of family caregiving *The tension and interplay between experiencing both challenges and rewards with family caregiving*	Psychological and physical symptoms affected by family caregiving	“Sometimes I feel really lonely and bored because of taking care of my uncle almost every day” (P2G5).
	Care recipient disease progression	“I could actually recall some good memories when she was young and strong also when seeing how she’s not able to move on their own and also forgetting my name, forgetting, who I am, that I’m her son” (P2G1).
	Lack of time for self	“I noticed that taking care of my aunt restricts me from having my much-needed free time and it’s hard, taking care of myself and catering [to] someone [who] has more needs than me. I am sapped of sleep time, rest time and free time” (P3G5).
	Fulfilling a sense of duty	“Because she was there. She was my biggest support and I feel like doing this for her is like the biggest gift ever I could give to her” (P6G2).
	Closeness with care recipient	“Seeing him happy and interacting with my kids. As much as it’s hard, when he thrives, it’s good” (P1G2).
	Altruism/fulfillment	“It kind of makes me feel unique and gives me a positive type of pride to know that I do something a lot of people would struggle with” (P1G5).
Coping strategies *The strategies caregivers deploy to cope with life and caregiving demands.*	Personalized self-care strategies	“I don’t really have anything else besides hanging out with my kids, you know” (P1G2). “I try to maintain my mental and emotional health. Watching movies, I watch movies a lot and listen to music too” (P5G2). “Doing yoga and listening to some of my favorite music, it’s an escape for me” (P6G2).
	Social connection	“I try to spend time with friends and do relaxing things like watching a movie or go for a walk” (P1G5).
	Humor and positive emotions	“I like to laugh out most of the pain. I do watch a lot of cartoons. So, when I do watch cartoons, I feel I laugh out my pain” (P2G1).
	Rest/respite/recharge	“Honestly, oddly enough, when I go to chemo and I have those moments to sit quietly. It helps. That’s my moment to sit quietly and just be” (P2G4).

## Results

Twenty participants identified as women (69%) and nine identified as men (31%). Participants had a mean age of 31 (*SD* = 4.10) with most (*n* = 28, 96%) caring for an adult (sibling/parent/grandparent), and one caring for children with chronic conditions (4%). Qualitative results with overarching themes, main themes, and sub-themes are outlined below and in Table 2. Quotes are denoted with participant number and focus group or interview number (P#G#).

There were two overarching themes, the Latinx Experience and being a “Super Caregiver,” and six main themes: (a) Family Well-Being; (b) Occupational and Financial Well-Being; (c) Social Support Dynamics; (d) Challenges of Family Caregiving; (e) Rewards of Family Caregiving; and (f) Coping Strategies. In discussing the Latinx experience, caregivers described having immigrant experiences: “usually we go and help out here at the border . . . donations, helping people find placement” (P1G2). Threaded throughout conversations of family caregiving, they described the importance of family and their community: “It was a good thing I could meet people from my experience in my community” (P1I1). Caregivers also noted the difficulty of navigating barriers in the U.S. system as a minoritized individual: “I feel like we minorities should get some conversation . . . She [mother with diabetes] deserves better care” (P1I1). When describing being a “Super Caregiver,” the need to do it all was emphasized: “Normally we are givers and we always feel we’ve never given enough” (P4G3).

### Family Well-Being

During discussions of family caregiving responsibilities, Latinx family caregivers emphasized family well-being with the following sub-themes: (a) *importance of family relationships*, (b) *balancing family and caregiving, and* (c) *challenges maintaining intimate partner relationships.* In addressing t*he importance of family relationships* one caregiver noted: “I just want my grandma to have someone because she’s alone, at home, most of the time, so I just want her . . . to not feel that loneliness so much” (P5G2). With this prioritization came the need to *balance their time and attention with family and caregiving*. One participant described, “[The] most difficult experience for me was time management. I had to give attention as needed to my dad, my husband, and to my kids. It’s hard to balance out the time” (P6G3). In addition, some caregivers described *challenges maintaining intimate partner relationships*: “. . . we’ve also had this argument at home. My husband telling me I have more time with my dad. That I’m not home with the family” (P3G2).

### Occupational and Financial Well-Being

The difficulties of balancing employment with caregiving and the impact of family caregiving on financial well-being were frequently noted by Latinx Millennial caregivers. The following sub-themes addressed work challenges: (a) *presenteeism, absenteeism, leaving the workforce, and* (b) *workplace support and flexibility*. Caregivers identified issues related to *presenteeism*: “I do have some challenges sleeping and also sleep at work” (P2G1); *absenteeism*: “If I have to come late, and sometimes I would turn to my co-workers to cover for me. I usually pay them for it” (P1I1); and *leaving the workforce*: “I worked as a sales representative at a restaurant before I had to quit my job” (P6G3). Some caregivers described having *workplace support and flexibility*, with some having flexibility to work from home, “I’m a teacher, but I am currently remote teaching. Well, you know I get to stay at home . . . I have my kids, my grandfather . . . he has dementia” (P1G2). One caregiver identified support from co-workers: “My friends at my workplace sometimes cover my shift for me, so I can go take care of my aunt” (P3G3). Latinx Millennial caregivers described financial challenges in addition to occupational challenges with the following sub-themes: (c) *cost of medication and healthcare*, (d) *balancing living costs with caregiving, and* (e) *the stress of daily living costs.* In describing the *cost of medication and healthcare* for the care recipient, one caregiver stated, “I do not have a job to pay insurance fees and everything so it really hasn’t been easy for me” (P5G3). In *balancing living costs with caregiving*, a caregiver simply replied, “Money management is one of the most difficult [parts of caregiving]” (P4G3). Finally, in addressing *daily living costs*, caregivers described experiencing a stretched income and few resources, “You know, my income is very low” (P3G1).

### Social Support Dynamics

Latinx Millennial caregivers identified the dynamics of social support, with both needing and receiving social support in diverse ways. Sub-themes addressing this included: (a) *structural support*, (b) *family support*, (c) *connections with other caregivers*, (d) *specific support needed: educational support, emotional support, and financial support.* In addressing *structural support*, caregivers described needing it from the government: “I know we would feel better supported if we had more financial support and kind of had a recognition from the government” (P1G5); community resources and settings: “My son’s schoolteachers are not really supportive. Sometimes I might be late to pick up my son, the teachers tend to shout at me” (P5G3); and the healthcare system: “Getting the health industry to help us is really hard nowadays” (P3G4). Some caregivers felt they did receive *family support*, “. . . me and my partner share responsibilities. She’s in charge of the kids while I’m in charge of my grandma” (P2G1), while others noted less *family support*, “I have a few siblings and I think I would really appreciate if they supported me in caring for my dad” (P1G5). Caregivers described needing *connections with other caregivers*: “It would be nice knowing that there are other people like me, too, and we could share useful info and advice” (P3G5). They described additional areas of support, either needed or received, including *financial*: “I have four siblings . . . I do expect a lot of financial assistance from them” (P5G2); *emotional*: “. . . emotional support because I didn’t know the emotional and mental drain it’s going to put on me” (P2G4); and *educational*: “It would be really helpful knowing more about an illness or issue” (P3G5).

### Challenges and Rewards of Caregiving

Latinx Millennial caregivers also noted the tension of experiencing both challenges and rewards from their family caregiving with the following sub-themes: (a) *psychological and physical symptoms affected by family caregiving*, (b) *care recipient disease progression*, (c) *lack of time for self*, (d) *fulfilling a sense of duty*, (e) *closeness with care recipient, and* (f) *altruism/fulfillment*. In describing e*xperiencing psychological or physical symptoms related to their caregiving*, one caregiver stated: “I feel down, I feel moody and I can’t close my eyes properly to get some sleep” (P2G1). Caregivers noted the painful process of *watching disease progression in the care recipient*: “it is a bit depressing when I see my dad in that state” (P8G3). A large concern shared by many caregivers was *lack of time for themselves*: “I don’t really care how good you are at this . . . I’m not able to have time for myself” (P1G1). Despite the challenges related to family caregiving, caregivers described clear rewards. One caregiver described their duty to their child as an advocate: “I am my kid’s best advocate as a special school nurse. I’m already in the know of what is going on for services for kids with autism” (P9G3). Another caregiver described the *closeness* developed through caregiving, “You create this kind of bond between the two” (P4G3). In addressing *fulfillment and altruism*, a caregiver noted, “I’m so glad that I was there to work with her at this phase” (P5G3).

### Coping Strategies

Latinx Millennial caregivers deployed diverse coping strategies including: (a) *personalized self-care strategies*, (b) *social connection*, (c) *humor and positive emotions, and* (d) *rest/respite/recharge*. Caregivers described engaging in *personalized self-care strategies* such as naps, listening to music, having quiet time, yoga/meditation, reading, and watching movies: “doing yoga and listening to music is just everything for me” (P6G2). Caregivers identified *social connection* as a meaningful source of coping: “[I] get someone to talk to about this kind of thing. I’m really happy that I’m able to share this with some people” (P3G4). Several caregivers described focusing on *humor and positive emotions*: “It’s helpful to go back . . . and search my head for the good things that happened and made me happy” (P1G5). An area of coping that was fairly universal for these caregivers was having time to *rest, obtain respite, and recharge*: “Let me just walk outside, let me go ahead and just kind of breathe for a moment” (P1G2).

## Discussion

Findings from this study highlighted the distinct issues navigated by Latinx Millennial caregivers due to both cultural and social contexts as well as the life stage with multiple family responsibilities and job demands. The idea of being a “super caregiver” was described, where caregivers felt that they had to do it all. In addition, the focus on family, community, and social connection was particularly emphasized among these caregivers.

Caregivers described experiences related to their Latinx identity, including immigration experiences, cultural norms of family and community, and decreased access to resources. Caregiving cannot be separated from social context, systems of power, and the other roles and identities caregivers enact (Aaron et al., 2022; Crenshaw, 1991; McCauley et al., 2018). For example, Latinx caregivers provide more care and contribute more financially with caregiving than White counterparts (NAC & AARP, 2020). Significantly, while doing so, these caregivers report barriers to accessing societal support (Mage et al., 2024). Some well-known systemic barriers for the Latinx community include language access, built environments that do not facilitate access to food, community resources, and healthcare, provider bias, immigration issues, decreased insurance and healthcare access, and history of abuse from the healthcare system (Santana et al., 2023).

Many caregivers in our sample, both men and women, described the need to be a “super caregiver,” where they were meeting the demands of work, intimate relationships, parenting, other family, friends, and family caregiving, which has been echoed in other research studies (Mendez-Luck, John Geldhof, et al., 2016). Other study themes of family and occupational well-being align with this idea of balancing many demands and is supported by work from Badger et al. (2023) that noted higher caregiver burden in Latinx caregivers for finances, family, and schedules. In addition, Martinez and Gonzalez (2022) described a perspective of exceptionalism in the way care is provided in the Latinx community, thereby delaying support from community services. Our sample of both Latinx men and women described more caring attributes, intentions, and outcomes that align with caballerismo and marianismo, especially when describing the rewards of caregiving such as fulfilling a duty, closeness with the care recipient, and a sense of fulfillment (Arciniega et al., 2008). This is different from research in middle-aged and older caregivers who emphasized machismo and marianismo (Jaldin et al., 2023). Yet, the cost of being a super caregiver in this study was development of physical/emotional symptoms related to family caregiving and a lack of time for self. This tension of both challenges and rewards for caregiving is reflected in other research with Latinx family caregivers (Martinez & Gonzalez, 2022).

Participants in this study reported feelings of pride, closeness, and fulfillment related to caregiving. While familismo provides a sense of community and belonging, it may also influence caregivers to prioritize the family above the unique developmental milestones Millennial Latinx caregivers are navigating in their lives, such as career and financial security, intimate relationships, and parenting (Badger et al., 2019; Brooks et al., 2014; Thomas Hebdon et al., 2022). This could be a source of stress and ambivalence as they navigate the sense of duty in family caregiving while also grappling the burden and challenges that are inherent to family caregiving (Gelman, 2014; Guidi et al., 2021). This may also speak of the pattern of Latinx family caregivers providing care in the home versus institutionalized settings, an experience echoed by many participants and other research (Crist et al., 2009). Despite these patterns, there can be institutional neglect in providing and offering services that are culturally congruent to the needs of Latinx Millennial family caregivers (Martinez et al., 2022).

### Clinical Implications

For nurses supporting Latinx Millennial family caregivers, it is key to acknowledge individual experiences and social contexts (Krogstad et al., 2023; National Alliance on Mental Illness, 2023). For example, caregivers in this study demonstrated several methods of adaptive coping including self-directed activities, social connection, and rest. Nurses can provide resources to caregivers that enhance what caregivers are already doing to cope with the burden of caregiving (Guidi et al., 2021). In the study, caregivers described the use of music, quiet moments, and meditation for coping, which are strategies that nurses can facilitate to support emotional and spiritual well-being (de Diego-Cordero et al., 2022). The need for rest and respite is a recurring theme across caregiver age and experience (NAC & AARP, 2020). While clinical and research interventions may be able to target this, nurses can do upstream work and health policy advocacy to provide respite and support services for these caregivers. When considering coping or respite interventions for Latinx Millennial caregivers, accessibility, timing, disruptiveness, and family preferences for home- or institutional-based care need to be considered (Crist et al., 2009; Martinez et al., 2022; NAC, 2019; Thomas Hebdon et al., 2022).

### Limitations

A main limitation of this study was the exclusion of individuals who did not have technology to accommodate Zoom meetings. This could have contributed to greater socioeconomic homogeneity with the study sample. In addition, there were more study participants who were women, while 57% of Latinx Millennial caregivers are men (Flinn, 2018). A sample that is representative of the gender and socioeconomic distribution of Latinx Millennial caregivers is needed to understand how gender, socioeconomics, culture, life stage, generational cohort, and family caregiving impact each other.

## Conclusion

In this preliminary study examining the experiences and needs of Latinx Millennial caregivers, the importance of family and community as a motivation for caregiving, and the tension between family and personal needs are key to understanding this caregiving group. In addition, Latinx Millennial caregiver experiences with immigration and resource access issues are of particular concern due to structural inequities in the U.S. Finally, being a super caregiver was highlighted with the need to be everything for everyone, which may place undue stress on these family caregivers. These insights can guide future research and understanding of this important caregiving group and may provide intervention targets to address Latinx Millennial caregivers’ strengths and unmet needs.
